# Programmatic options for monitoring malaria in elimination settings: easy access group surveys to investigate *Plasmodium falciparum* epidemiology in two regions with differing endemicity in Haiti

**DOI:** 10.1186/s12916-020-01611-z

**Published:** 2020-06-23

**Authors:** Thomas Druetz, Gillian Stresman, Ruth A. Ashton, Lotus L. van den Hoogen, Vena Joseph, Carl Fayette, Frank Monestime, Karen E. Hamre, Michelle A. Chang, Jean F. Lemoine, Chris Drakeley, Thomas P. Eisele

**Affiliations:** 1 Center for Applied Malaria Research and Evaluation, Department of Tropical Medicine, Tulane University School of Public Health and Tropical Medicine, New Orleans, LA USA; 2 Department of Social and Preventive Medicine, School of Public Health, University of Montreal, Montreal, QC Canada; 3 Department of Infection Biology, London School of Hygiene & Tropical Medicine, London, WC1E 7HT UK; 4 Malaria Zero Alliance, CDC Foundation, Port-Au-Prince, Haiti; 5 IMA World Health, Port-au-Prince, Haiti; 6 Malaria Branch, Division of Parasitic Diseases and Malaria, Center for Global Health, Centers for Disease Control and Prevention, Atlanta, GA USA; 7 CDC Foundation, Atlanta, GA USA; 8 Programme National de Contrôle de la Malaria, Ministère de la Santé Publique et de la Population (MSPP), Port-au-Prince, Haiti

**Keywords:** Convenience sample, *Plasmodium falciparum*, Epidemiology, Surveillance

## Abstract

**Background:**

As in most eliminating countries, malaria transmission is highly focal in Haiti. More granular information, including identifying asymptomatic infections, is needed to inform programmatic efforts, monitor intervention effectiveness, and identify remaining foci. Easy access group (EAG) surveys can supplement routine surveillance with more granular information on malaria in a programmatically tractable way. This study assessed how and which type of venue for EAG surveys can improve understanding malaria epidemiology in two regions with different transmission profiles.

**Methods:**

EAG surveys were conducted within the departments of Artibonite and Grand’Anse (Haiti), in regions with different levels of transmission intensity. Surveys were conducted in three venue types: primary schools, health facilities, and churches. The sampling approach varied accordingly. Individuals present at the venues at the time of the survey were eligible whether they presented malaria symptoms or not. The participants completed a questionnaire and were tested for *Plasmodium falciparum* by a highly sensitive rapid diagnostic test (hsRDT). Factors associated with hsRDT positivity were assessed by negative binomial random-effects regression models.

**Results:**

Overall, 11,029 individuals were sampled across 39 venues in Artibonite and 41 in Grand’Anse. The targeted sample size per venue type (2100 in Artibonite and 2500 in Grand’Anse) was reached except for the churches in Artibonite, where some attendees left the venue before they could be approached or enrolled. Refusal rate and drop-out rate were < 1%. In total, 50/6003 (0.8%) and 355/5026 (7.1%) sampled individuals were hsRDT positive in Artibonite and Grand’Anse, respectively. Over half of all infections in both regions were identified at health facilities. Being male and having a current or reported fever in the previous 2 weeks were consistently identified with increased odds of being hsRDT positive.

**Conclusions:**

Surveys in churches were problematic because of logistical and recruitment issues. However, EAG surveys in health facilities and primary schools provided granular information about malaria burden within two departments in Haiti. The EAG surveys were able to identify residual foci of transmission that were missed by recent national surveys. Non-care seeking and/or asymptomatic malaria infections can be identified in this alternative surveillance tool, facilitating data-driven decision-making for improved targeting of interventions.

## Background

Haiti and the Dominican Republic are committed to eliminating malaria [1–3]. The island they share (Hispaniola) remains the only one in the Caribbean with endemic malaria transmission, with the majority of cases being reported in Haiti [2, 4]. The predominant malaria parasite in Haiti is *Plasmodium falciparum*, and the main malaria vector is *Anopheles albimanus*, which has a tendency to bite and rest outdoors [5–7]. Household surveys were conducted in Haiti at the national level in 2011, 2012, and 2015, and each national survey consistently measured parasite prevalence at < 1% by polymerase chain reaction (PCR) [5, 8]. Elimination feasibility is enhanced by the absence of chloroquine-resistant *P. falciparum*, which is still used as a first-line malaria treatment, and the absence of pyrethroid resistance in *An. albimanus* [7, 9–14].

As in most eliminating countries, malaria transmission is highly focal in Haiti [15, 16]. In this context, programmatic efforts need to be reoriented to identify specific reservoirs of infection (both geographically defined areas and/or high-risk populations) and to monitor the effectiveness of targeted interventions to disrupt malaria transmission in these foci [17]. However, household cluster surveys that are typically conducted to estimate malaria prevalence are not suitable in low and focal transmission settings: the intensive resources (time, money, logistics, etc.) required to achieve sufficient statistical precision in settings with low parasite prevalence and variations across space and time become inefficient and programmatically untenable [18, 19]. Health facility-based passive surveillance data have limited capacity to identify transmission foci when confirmed cases are not able to be traced back to the household, access to healthcare is low, and data quality issues are common. Furthermore, many infections are expected to be asymptomatic; these individuals do not seek care at health facilities and usually remain in the community undetected [20, 21].

A promising alternative for malaria control programs to supplement passive surveillance is to conduct surveys of populations within easily accessible venues, commonly referred to as easy access group (EAG) surveys [22–25]. Logistical issues and costs are considerably reduced in EAG surveys as compared to population-based household surveys, and they have proven to be effective proxies for assessing burden and transmission intensity (using diverse metrics: parasite rate, incidence rate, seroconversion rate) in the community and for measuring the effectiveness of malaria interventions [19, 26]. However, as pointed out in a recent systematic review, EAG studies have all been conducted in moderate or high malaria transmission settings [27]. In addition, the literature has focused on assessing and correcting for the inherent selection bias in EAG surveys instead of the programmatic relevance of the approach [24, 27, 28].

The aim of this study was to assess if, and if so, how and which type of EAG surveys can improve understanding malaria epidemiology, identify residual foci in two regions with different transmission profiles, and inform programmatic decision-making and intervention targeting within Haiti. Different types of EAG venues including churches, primary schools, and health facilities were investigated.

## Methods

### Site selection, sampling, and consent

**Fig. 1:**
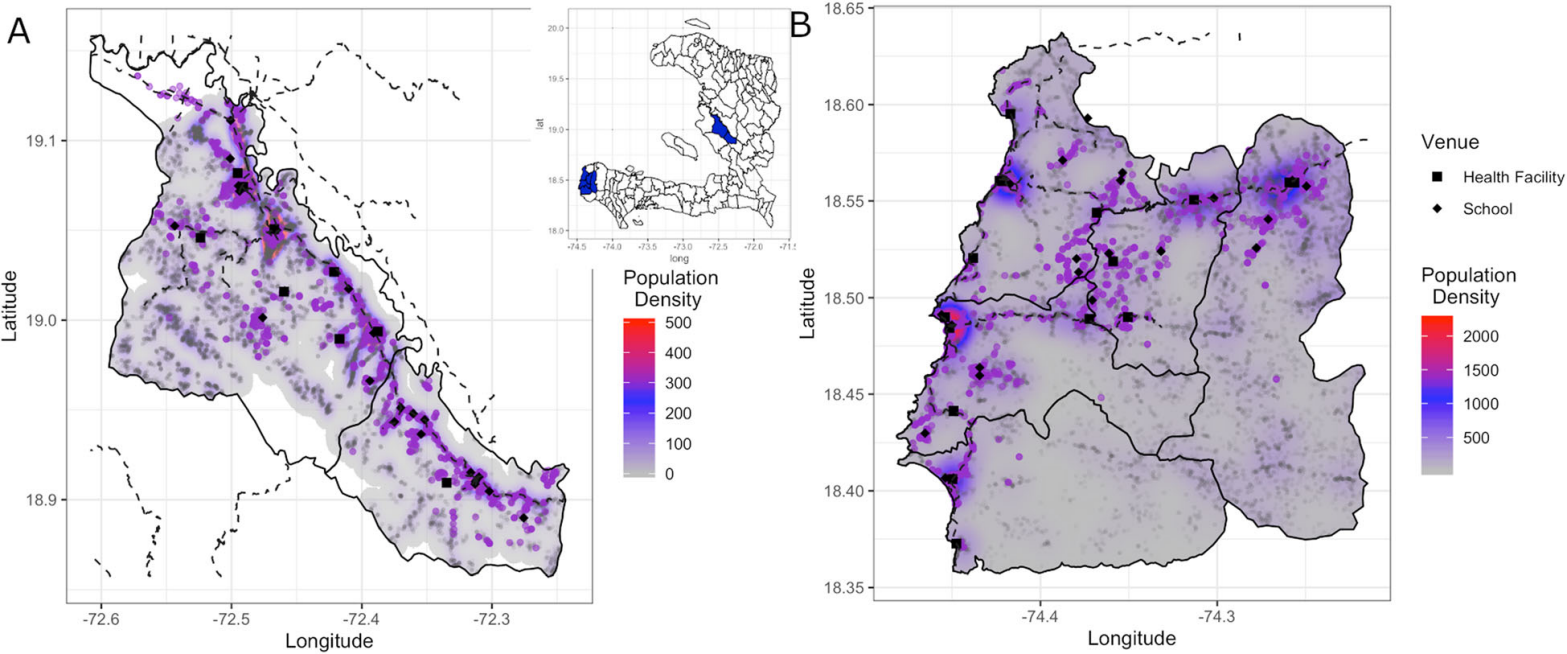
The population distribution in Artibonite (**a**) and Grand’Anse (**b**) study regions shown as a heatmap (with the structure locations shown as light gray dots) with the location of those sampled during the EAG surveys overlaid as purple dots. The locations of the health facilities (square) and schools (diamond) where sampling occurred are also shown with the location of the study regions within Haiti shown in blue in the inset map

This study was conducted in 2017 in rural Haiti. Two sets of EAG surveys were performed. The first took place in May/June in the Artibonite Valley of central Haiti, with the second in October/November in the Grand’Anse department, south-western Haiti (Fig. 1). These periods correspond to the two annual transmission peaks in Haiti [29]. The study region comprised areas in two communes in Artibonite (Fig. 1a) and five communes in Grand’Anse (Fig. 1b), with total estimated populations of 138,032 and 156,138, respectively [30]. The total population for the Artibonite study region was obtained from a concurrent census conducted in the area, whereas for Grand’Anse, the most complete post-hurricane data source was obtained via Open Street Map (www.openstreetmap.org)—a category 5 hurricane (Matthew) hit Grand’Anse in October 2016 and severely impacted the habitat. The two contrasted regions were purposively selected in collaboration with the National Malaria Control Program (PNCM) due to the relatively higher malaria incidence in these communes compared to the rest of Haiti; in 2016, national passive surveillance suggested that the annual incidence rate was 3 per 1000 in the selected Artibonite communes and 27 per 1000 in the Grand’Anse communes. Mean malaria incidence across Haiti is < 2 per 1000. Both regions are characterized by mountainous terrain with intersecting rivers and valleys, with Artibonite being landlocked and Grand’Anse along the Caribbean Sea coast. They encompass rural, semi-rural, and urban populations—the few largest towns in the study region all have < 20,000 inhabitants.

The EAG venue types tested included primary schools, health facilities, and churches. Informal interviews suggested that schools and churches are some of the most common gathering places in Haiti and among the few venues in remote and rural areas expected to be spatially representative while having sufficient attendance to provide information about the local population. Health facilities also serve as a convenient sentinel population with periodic all-attendee surveys providing more granular information while being easily integrated into the general public health system [20]. The selection procedures for each are described below in turn and summarized in Table 1, with any methodological differences between the two regions highlighted.

**Table 1:** Overview of venue and participant selection procedures for each for the venue types

	Primary school	Health facility	Churches
Region venue sampled	Artibonite; Grand’Anse	Artibonite; Grand’Anse	Artibonite
Venue			
Sampling frame	Government and non-governmental	Government and non-governmental	Convenience census
Inclusion criteria	At least 100 pupils registered	Staffed daily with clinician attending patients	At least 100 regular attendees
Sampling strategy	- Stratified random sample according to remoteness measured as distance to main roads - *N* = 21 (Artibonite); *N* = 25 (Grand’Anse)	- Census of all functioning facilities - *N* = 9 (Artibonite); *N* = 16 (Grand’Anse)	- Stratified random sample by sub-commune and denomination - *N* = 9 (Artibonite)
Participant			
Target sample size	- 150 pupils per school	- A total of 450 individuals per facility - 150 individuals per each age category (6 months to 5 years; 6–15 years; > 15 years)	- All attendees
Sampling strategy	- Pupils in class 2 to 6 - 25 pupils per class selected by random number table	- All patients attending the health facility above 6 months of age not requiring urgent medical attention - All people accompanying patients above 6 months of age	- Above 6 months of age - Attended the church service
Consent	- Written from school director - Community parental meetings, with opt-out consent - Assent from children	- Written informed consent - Written assent for children 7 to 17 years old	- Written informed consent - Written assent for children 7 to 17 years old
Geolocation strategy	- All hsRDT-positive individuals by handheld GPS - Random selection of 30% of people testing hsRDT negative by GPS trackers and/or handheld GPS	- All coordinates collected by handheld GPS - All hsRDT-positive individuals - Random selection of 30% of people testing hsRDT negative	- None

#### Primary schools

A census of all primary schools, including public, private, and faith-based institutions, was conducted (Artibonite, *n* = 234; Grand’Anse *n* = 144) to provide a sampling frame. Information collected included the Global Positioning System (GPS) coordinates of the school, number of registered pupils for the current school year, and contact details. A sample of 21 (Artibonite) or 25 (Grand’Anse) schools with at least 100 enrolled pupils was selected in each region using a stratified random sampling procedure to ensure equal distribution across sub-communes and by remoteness (defined as close or far from main roads according to Euclidean distance). At each school, a maximum of 25 pupils per grade, in grades 2 to 6, were selected to ensure a broad age range, with a maximum of 150 pupils included per school [26]. School children from the same household were gathered, even if they were in different grades, in order for the oldest siblings to assist their younger sibling(s) in answering the questionnaire.

Informed consent for sampling of school children used opt-out methods. Written informed consent was obtained from each school director, after consultations and consent from the Department of Education and local leaders. Community meetings were held at each school 1 week before the survey day to explain study objectives and procedures to the parents and to respond to any questions. Parents maintained the right to withdraw their children from the study at any time. Parents wishing to opt-out either asked their child to refuse if selected for sampling or informed the school director to ensure their child did not participate. Finally, written or thumbprint assent (countersigned by an adult witness in the latter case) was sought from children above age 6 years after having the study and procedures explained. Children who refused to participate were replaced by another child from the same grade.

#### Health facilities

A list of all functioning (i.e., with attached health personnel) public and private health facilities in each study region was obtained from the Ministry of Public Health and Population and checked for completeness and accuracy. All functioning health facilities in the study regions (Artibonite = 9; Grand’Anse = 16) were included in the survey. All individuals attending the facility during the study period, as well as anyone accompanying them (e.g., parents of a sick child), were eligible for inclusion. Those under 6 months of age, having had previously visited the facility during the study period, attending a scheduled treatment clinic (e.g., antenatal care, HIV), or requiring urgent care were excluded. At each facility, a maximum of 150 people from each of three age categories (6 months to 5 years, 6–15 years, > 15 years) were targeted, following the protocols described in Stresman et al. [20]. Informed written consent was sought from all adult participants and from the parent/guardian of all participating children (< 18 years). In addition, written assent was obtained from those between the ages of 7 and 17. Minors who were married, were pregnant, had children, or were the head of the household were considered mature minors and consented as adults. Thumbprint consent or assent (countersigned by a witness) was used for illiterate participants.

#### Churches

Except for Catholic churches, no comprehensive list of churches was available, so a convenience census was conducted by visiting the area with a local guide and by asking community members to target those likely to have at least 100 regular attendees (*n* = 83). The GPS coordinates, denomination, estimated number of regular attendees, and contact details were collected. Nine churches were selected from the list generated as part of the convenience census using a stratified random sample by sub-commune and denomination. This approach was employed due to logistical constraints: sampling was limited to weekends, with Adventists congregating on Saturdays and Catholics and Protestants on Sundays. All those aged over 6 months who attended the service were eligible for inclusion. There was no limitation on the maximum number of participants included per church. Informed consent and assent procedures followed those described above for health facilities. Churches were no longer included in the study in Grand’Anse due to the logistical challenges in obtaining a sampling frame and the large number of people to sample in a short period.

### Survey procedures

All consenting survey participants completed a questionnaire on a mobile data collection platform (CommCare, Dimagi, Cambridge, MA) on an Android tablet (Blu Studio 7.0 Phablet, Miami, FL). Questionnaire data were automatically pushed to a secure cloud-based server using the local mobile phone network. Questions included age, sex, how they traveled to the venue, history of fever in the past 2 weeks, treatment-seeking behaviors, travel history, and any vector control methods used in the home. Malaria risk associated with travel was ascertained using the self-reported communal section (smallest administrative unit) of their destination. The malaria burden associated with the reported travel destination was described as high (any cases reported) or low (no cases reported), according to the number of cases reported by the Ministry of Health surveillance system (District Health Information System (DHIS2)). Burden within the study site was dichotomized into high and low burden communal sections and was grouped according to highly sensitive RDT (hsRDT) prevalence being greater or lesser than 10%. Reported bednet ownership and use the night before the survey were also collected. Current fever was defined as those with an axillary temperature greater than 37.5 °C. The questionnaire for school children was simplified to ensure it was age appropriate.

All participants were asked to provide a finger-prick capillary blood sample to test for a *P. falciparum* parasite infection. Malaria parasite infections were detected using a conventional histidine-rich protein 2 (HRP2)-based rapid diagnostic test (cRDT; SD Bioline Ag. Pf, Suwon City, South Korea) and a hsRDT (SD Bioline Ag. Pf prototype, Suwon City, South Korea). Individuals found to be positive by the cRDT were provided treatment according to the national guidelines in Haiti, which combines a 3-day course of chloroquine and a single low dose of primaquine. The hsRDT results were the primary outcome of this study, but did not inform treatment as the hsRDTs are only approved for investigational use in Haiti. The intention of performing both tests was to contribute to a multi-site study that aimed to assess the hsRDT performance compared to the cRDT—results have been published elsewhere [31]. Treatment was administered by the study team in schools and churches and by clinical staff in health facilities.

In schools, children with positive cRDT were traced to their home at the end of the day, where a member of the study team provided the drugs to the parents or caretaker and advised them on how to administer the course to the child. In schools and health facilities, all participants testing positive by either type of RDT and a random selection of 30% of those testing negative by both RDTs were traced to their household. At the household, spatial coordinates were obtained with a handheld GPS device (Garmin, Olathe, KS). In primary schools, household location was obtained using a wearable GPS tracker for an additional randomly selected subset of 30% of individuals, irrespective of RDT status or whether they were also selected for GPS tracking, where the spatial location of the household was extracted based on the nighttime location of the participants.

### Statistical analysis

All statistical analyses were conducted in open-source R statistical software V3.5.2 (R Foundation for Statistical Computing, Vienna, Austria). Malaria foci were identified by visual inspection of the hsRDT malaria prevalence by venue and the number of hsRDT positives per household within the subset of individuals for whom coordinates were available. Active foci were therefore conceptualized as circumscribed areas—within a malarious region—that sustain malaria transmission, as per WHO’s definition [32]. Mixed effects regression models (lme4 package) were used to identify factors associated with being hsRDT positive using a negative binomial fit for Artibonite and Grand’Anse school data and a logistic fit for Grand’Anse health facility data. Models were fitted by maximum likelihood using a Laplace approximation [33]. Mixed effects were included to account for the region with individual-level information included as fixed effects. The venue type was included as a fixed effect in Artibonite due to models failing to converge with both venue type and region included, likely associated with the few positive individuals detected in Artibonite. Variables included in the full model included venue type, sex, household bednet ownership and use, recent travel to areas that are high risk for malaria, mode of transport to arrive at the venue, and current or recent (past 2 weeks) fever. Recent travel was defined as spending at least one night outside of the commune of residence in the past 3 months. A backward step-wise model selection process was used with the best fitting model selected according to the Akaike information criterion values.

### Ethical approvals

The procedures for both study regions were approved by the National Bioethics Committee in Haiti (1516-30), the London School of Hygiene & Tropical Medicine Ethics Committee (103939), and the Tulane Institutional Review Board (795709). All participants provided informed written consent and/or assent, with parental consent for the school surveys using an opt-out process (described above) approved by all ethics committees. Participation in the study was not remunerated.

## Results

**Fig. 2:**
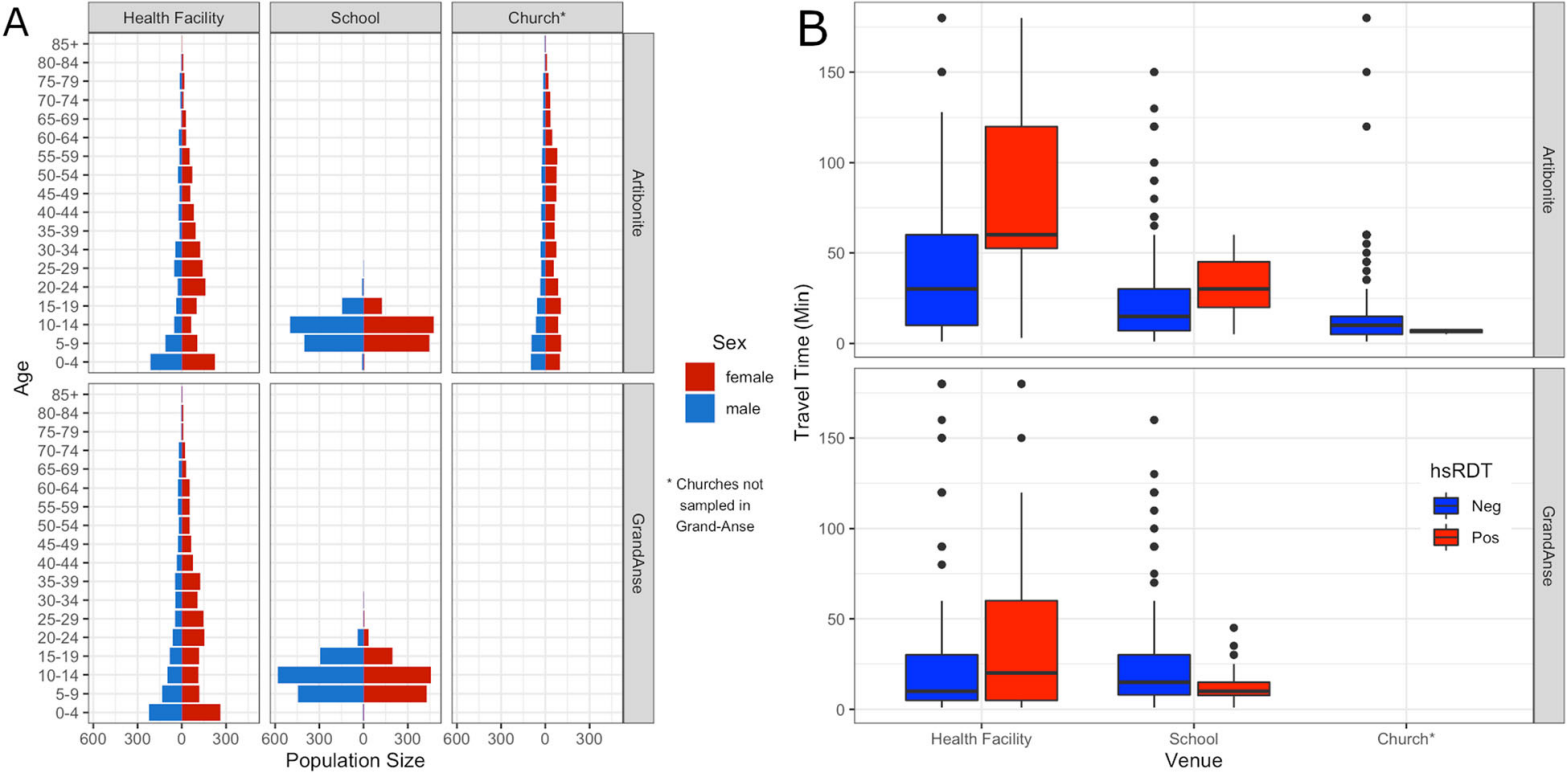
**a** Population pyramid with the age distribution of males (blue) and females (red) sampled shown per venue (columns) and study site (rows). **b** Self-reported travel time by venue types according to hsRDT positive (red) and negative (blue)

Overall, 11,029 individuals were sampled across the two study regions, with 6003 and 5026 participants across 39 and 41 venues in Artibonite and Grand’Anse, respectively (Table 2). The targeted sample size per venue type (2100 in Artibonite and 2500 in Grand’Anse) was reached except for the churches in Artibonite, where many attendees either left the venue before they could be approached or before the end of the consent process—unfortunately, it is not possible to report the exact number of these individuals. Refusal rate and drop-out rate among participants were < 1%, as was the proportion of participants who refused the test. Based on a visual assessment, the distribution of those sampled at the EAG venues was broadly reflective of the underlying population density where venues were sampled (Fig. 1). The median age of participants was similar between the regions for each venue type, but the age distribution of those sampled varied by venue types (Fig. 2a). More women were included in churches and health facilities, with an equal number of boys and girls sampled at schools in Artibonite, and more boys than girls at schools in Grand’Anse. Only 3.3% and 3.1% of individuals in Artibonite and Grand’Anse, respectively, reported recent travel, but this varied by venue types (Table 2). Reported bednet ownership was significantly lower in Artibonite (23.6%; 95% confidence interval [CI] 22.5–24.7%) compared to Grand’Anse (60.1%, 95% CI 58.6–61.4%, *p* < 0.0001).

**Table 2:** Summary and demographic info by venue type and department. The results are presented per venue as well as the range between clusters within each category

	Artibonite Valley						Grand’Anse			
	Health facilities		Primary schools		Churches		Health facilities		Primary schools	
	Value	Range	Value	Range	Value	Range	Value	Range	Value	Range
Venues—*N*	9	–	21	–	9	–	16	–	25	–
Sampled—*N*	2108	148–298	2126	20–150	1769	107–351	2521	119–196	2505	29–173
Median household size	5	1–18	5	1–20	5	1–16	5	1–20	6	2–17
Head of household occupation—%										
Shop keeper	38.7	20.1–47.1	19.6	1.4–34.7	30.5	23.3–41.0	27.5	0–61.2	11.3	0–36.4
Agriculture	34.4	26.9–53.3	63.2	28.0–95.8	42.4	24.2–60.1	47.9	15.3–97.8	77.6	30–100
Fisherman	0.05	0–0.5	0	0	0	0	3.8	0–26.4	1.6	0–10.0
Day laborer	5.5	0–13.8	5.4	0–16.7	3.9	0.8–11.2	4.1	0–16.0	2.7	0–12.0
Civil servant	3.7	0–11.4	3.5	0–12.5	4.3	0.8–11.2	2.2	0–8.7	1.6	0–8.2
Retired	3.5	0–10.0	2.0	0–7.3	4.6	0–6.7	2.0	0–8.8	1.1	0–17.2
Others	14.1	6.0–21.8	6.2	0–13.2	14.2	6.1–29.9	12.4	0.7–30.0	3.9	0–16.4
Sex—% F	66.6	59.4–71.2	49.8	29.7–66.9	65.5	57.2–69.6	62.4	52.7–70.3	45.1	31.9–63.8
Median age (IQR)	23 (6–39)	18–29	10 (8–13)	7–14	26 (10–50)	17–40	21 (6.5–38)	15.5–29.6	11 (8–14)	8–17
Own bednet*—%	19.0	10.8–26.5	30.8	2.1–52.7	21.1	7.2–41.1	69.7	50.6–89.9	56.4	15.0–85.9
Bednet use if own*—%	54.8	29.0–95.6	67.6	40.9–100	48.2	11.1–60.0	79.7	60.2–98.4	79.9	18.6–100
Recent travel*—%	4.0	0–6.9	0.4	0–2.7	6.0	0.5–13.1	6.3	0–18.0	0.04	0–0.9
Own cell phone*—%	58.7	29.7–75.1	–	–	44.6	21.3–71.4	46.4	10.6–71.4	–	–
Fever past 2 weeks*—%	22.7	10.1–34.8	5.5	0–11.7	8.0	2.9–16.8	25.1	0.6–53.0	1.4	0–3.6
Seek care if febrile*—%	71.2	20.3–93.4	46.6	0–1.0	45.3	20.0–71.9	15.7	00–66.7	22.2	0–1.0

*The *N* applied to these data removes observations with non-responses or do not know responses

### Spatial risk and malaria foci

**Fig. 3:**
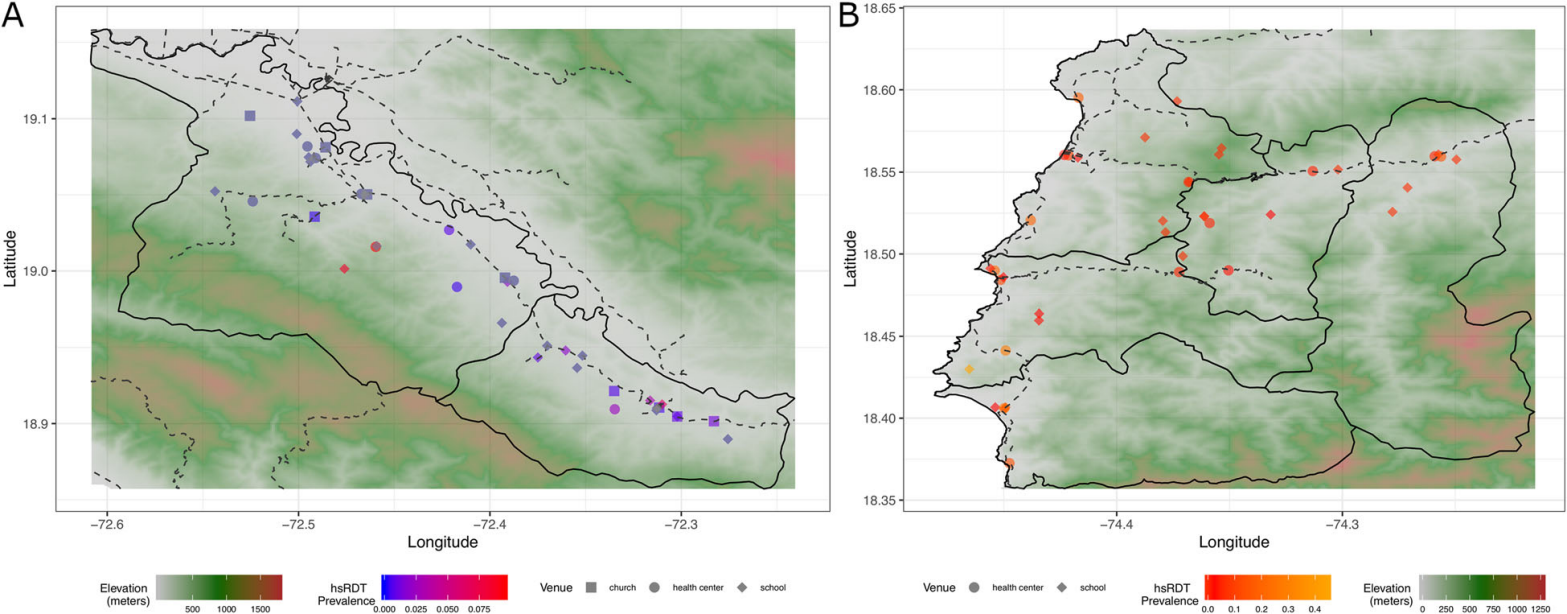
Maps showing the location of venues and shaded according to the overall hsRDT prevalence per venue and the elevation in meters for Artibonite (**a**) and Grand’Anse (**b**) EAG studies

Corresponding to the difference between the study regions in the underlying transmission, far more hsRDT-positive individuals were detected in Grand’Anse (355 hsRDT-positive individuals) as compared to Artibonite (50 hsRDT-positive individuals). The majority of infections in both regions were detected in health facilities (26/50 and 275/355 in Artibonite and Grand’Anse, respectively) with only six hsRDT positives out of 1769 individuals identified in churches in Artibonite. The hsRDT prevalence ranged between and across venues with the highest hsRDT prevalence in Artibonite detected at a health facility (9.5%) whereas in Grand’Anse, the highest burden was observed at a school (44.8%) (Table 3). Health facility- and school-level prevalence estimates led to the identification of similar foci of malaria burden in both regions, with the most prominent being in the center of the Artibonite study region in a mountain valley and along the coast in Grand’Anse (Fig. 3).

**Table 3:** hsRDT positivity according to demographic, by venue type and department. Cluster range shown is hsRDT pos/*N* tested

	Artibonite Valley						Grand’Anse			
	Health facilities		Primary schools		Churches		Health facilities		Primary schools	
	Value	Range	Value	Range	Value	Range	Value	Range	Value	Range
hsRDT pos *N* (%)	26 (1.2)	0–19 (0–9.5)	18 (0.8)	0–7 (0–8.5)	6 (0.3)	0–2 (0–1.2)	278 (11.0)	0–52 (0–27.9)	77 (3.1)	0–39 (0–44.8)
Age group *N* hsRDT pos (% *N*/*N* tested)										
< 5	0	–	0	–	1 (0.5)	0–3.1	33 (6.4)	0–27.3	0	–
5–15	3 (0.8)	0–10.5	16 (0.8)	0–9.5	1 (0.3)	0–2.9	110 (21.7)	0–60.6	71 (3.4)	0–44.8
> 15	23 (1.8)	0–11.3	2 (1.2)	0–16.7	4 (0.3)	0–1.2	135 (9.0)	0–22.9	6 (1.4)	0–25.0
hsRDT pos with current or recent fever *N* (% *N* febrile/*N* hsRDT pos)										
Yes	12 (50.0)	0–10.9	3 (16.7)	0–33.3	3 (50)	0–5.9	195 (70.9)	0–45.7	10 (13.0)	0–100
No	12 (50.0)	0–7.5	15 (83.3)	0–8.7	3 (50)	0–0.9	80 (29)	0–25.2	67 (87.0)	0–45.8
hsRDT pos with recent travel *N* (% *N* traveled/*N* hsRDT pos)										
Yes	22 (95.6)	0–8.3	0	–	0	–	10 (3.7)	0–33.3	0	–
No	1 (4.4)	0–8.7	18 (100)	0–8.5	6 (100)	0–1.3	260 (96.3)	0–28.1	73 (94.8)	0–44.2
Use of bednets—*N* hsRDT positive (% *N* bednet/*N* hsRDT pos)										
No bed net	21 (91.3)	0–10.9	15 (83.3)	0–8.7	5 (83.3)	0–1.4	82 (30.4)	0–38.6	42 (54.5)	0–42.6
Did not use a bednet despite possession	0	–	2 (11.1)	0–4.8	0	–	150 (55.5)	0–23.9	35 (45.5)	0–52.0
Use a bednet	2 (8.7)	0–10.5	1 (5.6)	0–33.3	1 (16.7)	0–7.7	38 (14.1)	0–50.0	0	–

**Fig. 4:**
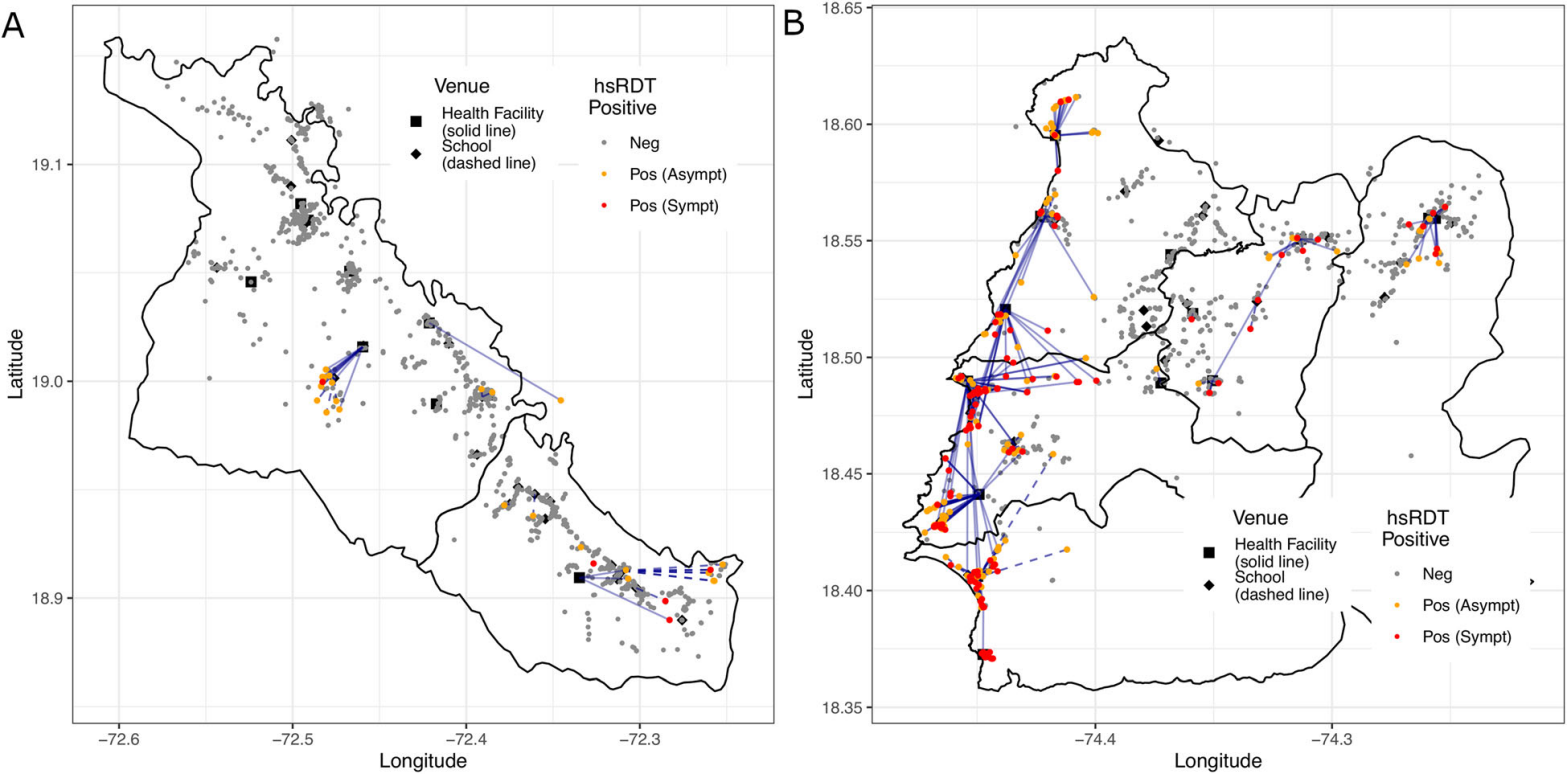
Maps showing the location of participants followed up with the households in **a** Artibonite and **b** Grand’Anse reporting negative (gray), positive but asymptomatic (orange), and positive but symptomatic (red) by hsRDT. Here, symptomatic is defined as a current temperature > 37 °C. Lines are shown connecting the household of individuals testing positive by hsRDT to the venue where they were sampled. The connecting lines are solid for those sampled at health facilities and dashed for schools

At the household level, further granularity becomes visible and enables a more precise delineation of venue catchment areas and sub-catchment delineation of foci boundaries. Based on the subset of individuals where household coordinates were available, the median distance traveled to a health facility was 2.35 km (interquartile range [IQR] 0.67–4.86 km) and 1.08 km (IQR 0.47–1.78 km) to go to school in Artibonite. In contrast, for the Grand’Anse region, the mean distance traveled to the venue was 1.26 km (IQR 0.68–1.71 km) for health facilities and 1.12 km (IQR 0.33–1.13 km) for schools. The mean distance traveled to the venue of those individuals testing positive for malaria by hsRDT was over 3 km and 1 km in Artibonite and Grand’Anse, respectively (Fig. 4; Additional file 1).

### Demographic characteristics of hsRDT-positive individuals

In Artibonite, the majority (29/50) of hsRDT-positive individuals were over 15 years of age. In contrast, 181/355 infections were detected in the 5- to 15-year-old age category in Grand’Anse (Table 3). The proportion of hsRDT-positive individuals who did not have a current or report a recent fever varied according to the region and the type of venue. In Artibonite, the proportion of asymptomatic infections (defined as no reported fever and current temperature < 37 °C) was 83% in schools and 50% in churches and health facilities. In Grand’Anse, the proportion was 83% in schools and 29% in health facilities. Across both regions, only 4 of 50 and 38 of 355 hsRDT-positive people in Artibonite and Grand’Anse, respectively, reported owning and sleeping under a bednet the previous night. Consistent with the actual distances reported above, in both Artibonite and Grand’Anse, those who were hsRDT positive reported longer travel time to attend the health facility than those who were hsRDT negative. This association was also observed among school children, but reached statistical significance only in Artibonite (Fig. 2b; see Additional file 2 for reported travel times by mode of transport).

The majority of the participants recruited at health facilities in Artibonite did not report recent travel, both among those testing positive by hsRDT (95.7%) and those testing negative (95.5%). Of those residing in Artibonite, the most common travel destinations were Port-au-Prince (*n* = 29), Saint-Marc (*n* = 20, medium-size city nearby), or Verrettes (*n* = 20; small city within the study region) with only eight participants visiting an area with high malaria risk. Similarly, in Grand’Anse, the vast majority of the participants recruited in health facilities did not report recent travel, both among those with a positive hsRDT (96.3%) and with a negative hsRDT (93.3%). The most common travel destinations of those residing in Grand’Anse included Port-au-Prince (*n* = 27), Jeremie (*n* = 23, medium-size city nearby), or Anse-d’Hainault (*n* = 16; small city within the study region), with 31 visiting an area with high malaria risk. Both in Artibonite and in Grand’Anse, the two most common reasons for travel were to visit friends and family (45% and 48%, respectively) and to engage in business (19% and 30%, respectively).

### Risk factors associated with hsRDT-positive individuals

Results of the mixed effects logistic regression model for the Artibonite study site suggest that males (adjusted odds ratio [AOR] = 1.96, 95% CI 1.07–3.59) and those with current or recent fever (AOR = 3.04, 95% CI 1.05–8.78) had significantly increased odds of being hsRDT positive, but there were no clear associations between sampling venue or commune of residence and hsRDT positivity in Artibonite (Table 4). Despite several individuals traveling to areas of high malaria risk either within or outside of the region where they reside, this was not associated with being hsRDT positive in this setting.

**Table 4:** Adjusted odds ratios (AOR) resulting from mixed effects regression models according to negative binomial for all venues combined in Artibonite and negative binomial for primary schools and logistic model for health facilities in Grand’Anse for hsRDT positivity, with the sampling venue as the random effect

Variable	AOR	95% CI	*p* value
Artibonite***			
Male	1.96	1.07–3.59	0.029
Fever*	3.04	1.05–8.78	0.040
Age (years)	1.02	1.01–1.04	0.012
**Grand’Anse**			
**Primary schools**			
Fever*	2.83	1.20–6.65	0.017
Male	1.65	0.99–2.73	0.051
Household size	1.10	0.99–1.22	0.067
Commune—low**	0.02	0.004–0.13	< 0.001
**Health facility**			
Fever*	1.26	1.21–1.31	< 0.001
Male	1.06	1.03–1.08	< 0.001
Commune—Low**	0.93	0.85–1.01	0.093

*Fever is defined as current fever (≥ 37.5 °C) or self-reported history of fever in the past 2 weeks

**Low burden communes defined by hsRDT positivity < 10% including Moron (2.4%), Dame Marie (5.4%), and Chambellan (1.6%) with Anse-d’Hainault (12.7%) and Les Irois (13.6%) being high burden

***Bednet ownership was included in the model due to its improving model fit (AIC), but the resulting estimate was not precise with a high *p* value so it was not included in the table (AOR 0.59, 95% CI 0.25–1.42, *p* value 0.238)

In Grand’Anse, travel was not significantly associated with hsRDT positivity (Table 4). In those sampled at schools, having current or recent fever (AOR = 2.83, 95% CI 1.20–6.65) was significantly associated with increased odds of being hsRDT positive, while being male (AOR = 1.65, 95% CI 0.99–2.73) and larger household size (AOR = 1.10, 95% CI 0.99–1.22) nearly reached statistical significance with a threshold of 95%. Living in one of the low-risk communes of Dame Marie, Moron, or Chambellan was associated with reduced odds of hsRDT infection (AOR = 0.02, 95% CI 0.004–0.13). Finally, in those sampled at health facilities in Grand’Anse, hsRDT positivity was associated with current or recent fever (AOR = 1.26, 95% CI 1.21–1.31) and being male (AOR = 1.06, 95% CI 1.03–1.08). Living in a low prevalence commune reduced the odds of being hsRDT positive (AOR = 0.93, 95% CI 0.85–1.01), although it did not reach statistical significance.

## Discussion

We implemented an EAG survey in the Artibonite and Grand’Anse regions of Haiti targeting health facilities, schools, and churches. This study is the first that used EAG surveys to investigate malaria transmission in a non-African country striving for elimination. Two regions with different levels of transmission intensity and expected local-level heterogeneity were assessed to explore this method’s suitability to different environments. Results suggest that surveys of individuals in schools and health facilities are acceptable within Haitian communities, are easy to conduct, and are able to identify some residual foci of malaria infection at the facility catchment and sub-catchment spatial scales. Surveys in churches were challenging to implement, due to the logistical difficulties associated with sampling large numbers of people in a short time frame and the few hsRDT-positive individuals identified, which limited the utility of the malaria information garnered. For these reasons, surveys were not conducted in churches during the second round of EAG surveys in Grand’Anse. Arguably, a qualitative study would be useful to assess (and compare) the acceptability and cost effectiveness of EAGs surveys in low malaria transmission settings.

In Artibonite, the only focus that was identified had been missed by the previous national surveys, which is unsurprising because they are not designed to detect confined foci in countries with low transmission. However, this particular focus was also missed by the passive surveillance system, which is unexpected since some cases were symptomatic and sought treatment at the nearest health facility. This illustrates the advantage of obtaining household-level spatial information as an add-on to venue-based sampling; it enabled sub-catchment identification of malaria foci. This is particularly relevant in the context of rural Haiti, where there are no residential addresses and where localities are vaguely defined—a reason why locating cases identified by passive surveillance is difficult without further investigation. The distance participants traveled to the venue suggests that assuming the venue reflects the surrounding area is not always true, and defining the venue catchment areas is important to accurately assign the spatial risk of transmission. Although the travel time question was less precise than physically tracing the household, the conclusions were consistent in suggesting the catchment was not restricted to the immediate area and provides a programmatic alternative if geolocating participants is not feasible. Importantly, the inclusion of spatial information identified sub-catchment malaria foci that would benefit from targeted interventions, confirming that the primary risk factor for being infected in this area is related to the location where participants lived. This finding is consistent with the notion of ongoing transmission in both study regions.

In this study, overall parasite infection prevalence (measured by hsRDT) was 0.8% in Artibonite, compared to 7.0% in Grand’Anse. The performance of the new hsRDT was only slightly better than that of cRDT (see Additional file 3), as was confirmed in a separate data analysis [31]. The spatial heterogeneity detected in this EAG study was missed by the nationally representative survey and by passive surveillance, to the extent that DHIS2 geolocation information does not go beyond communal section level. This study’s capacity was likely driven by a combination of the density of sampling that is feasible using the EAG approach, and for Grand’Anse specifically, potential changes in malaria transmission following Hurricane Matthew in October 2016 [34]. Altogether, this evidence suggests that targeting activities to the geographically clustered cases is warranted in Artibonite, where 60% of all hsRDT-positive cases were identified at two adjacent venues [35, 36]. By contrast, transmission is still relatively high in Grand’Anse, warranting broader and more intensive control interventions with any targeting focused on the coast [2, 3].

While it was not designed to be geographically representative of the population, this EAG study population broadly represented the distribution of the overall population where there was a venue included. The EAG results revealed malaria foci and therefore are a relevant strategy to inform decision-making and planning of interventions at the national and departmental levels as well as within health facility catchment areas. For example, the abovementioned focus in Artibonite was detected at an elevation of 650 m, while malaria transmission was thought to be limited to lowland areas under 500 m [4, 37]. Therefore, malaria interventions will need to be expanded to areas > 500 m where there is evidence of ongoing malaria infections.

While this study was not designed to assess malaria risk factors, it is noteworthy that there were very few variables associated in the models with greater odds of having a positive hsRDT among those included in the EAG surveys. Residing in a high burden commune was significantly associated with a higher odds ratio, but only for children recruited in schools in Grand’Anse. Arguably, this association could have been blurred in Artibonite, where the overall prevalence was lower and half of the cases were identified in a hotspot. These results suggest that as transmission decreases, malaria indicators at the smallest administrative unit commonly used for programs in Haiti (i.e., the commune level) could become less and less precise due to higher spatial heterogeneity. The presence of a current or recent fever was the only variable that was significantly associated with a higher risk of a malaria parasite infection in all populations studied. This association is not well-known by the Haitian population, as recently highlighted in a qualitative study [38]. Fever is often believed to be caused by a non-natural phenomenon and rarely prompts treatment-seeking through the formal health sector. However, it is important to underscore that, in health facilities, 46–57% of parasite positive detected by hsRDT in this study did not present with fever, depending on the study site. In schools, this proportion ranged 88–95%. This issue was recently discussed in a study that found a parasite prevalence of nearly 20% among asymptomatic women attending maternal clinics in Haiti—most were submicroscopic infections only detected by PCR analyses, and not by RDTs [39]. The present study adds to this evidence by suggesting that, in the Haitian regions with relatively higher incidence, *P. falciparum* infections can also be found in asymptomatic individuals at parasite density levels detectable by hsRDTs (~ 100 parasites/μl) [31]. New diagnostic strategies to address asymptomatic infections should be considered as part of any malaria elimination campaign in Haiti.

Contrary to many low-transmission settings, history of travel was not associated with increased odds of malaria infection in this study, which is consistent with the fact that travel concerned mainly trips to low-transmission areas [40]. While human mobility is generally (and excessively, some argue [41]) depicted as an obstacle to malaria elimination, in this setting, local movement may be sufficient to maintain transmission. Nevertheless, the elimination program may be supported by a better understanding of travel patterns in Haiti, notably patterns that we could not observe in the present study: daily movements, travel within communes, and travel patterns during holidays or for specific events (e.g., Carnival). Finally, male participants were at higher odds of malaria in Artibonite and in health facilities in Grand’Anse. Previous studies reported a higher malaria risk in males than in females in rural Haiti, although the difference was not statistically significant [15, 42]. Selection bias could partly explain this association, since adult women tend to visit health facilities more often than men (e.g., accompanying a sick child and for postnatal care visits). However, the association with sex remains even after controlling for the presence of fever. Other possible explanations are that men are more exposed to mosquito bites than women (because of their occupation, travel, or activities during nighttime) or are less exposed to preventive interventions such as bednet, intermittent preventive treatment, and treatment-seeking for fever). Because of the important repercussions that such a risk factor would have for programmatic decision-making, this association is currently being investigated by another study (Ashton et al., in preparation).

With the exception of churches, the surveys were easy to implement in EAGs. Preparatory work required a census of all health facilities and schools in the study area, with their GPS coordinates. This information was already available and was obtained thanks to the relevant governmental authorities. Therefore, contrary to household surveys, it was not necessary to perform a population census, nor to recruit participants by walking house-to-house. On the other hand, further actions were required to obtain spatial information of the participants’ residences. While several methods were tested in this study to collect this information, the most practical and easy way is likely to track the participants to their homes. If the EAG approach were to be used for programmatic purposes (e.g., surveillance, reactive case detection), it would imply tracking only participants with a positive RDT. In this study, community health workers or community members were easily trained and involved for this purpose. Finally, while several information procedures had to be followed in schools to recruit children without their parent’s direct consent, this requirement would not apply in a non-research context (i.e., if similar surveys were administered by the health authorities). Surveys can also be repeated in the same panel of EAGs for active surveillance or for impact evaluation of a specific intervention. The lapse that would be required between the different rounds of surveys will vary depending on several factors, notably the local malaria epidemiology and seasonality, the rationale of the study, and the characteristics of the intervention.

This study had some important limitations. By design, the samples obtained in these venues are not representative of the underlying population. Therefore, results cannot be directly extrapolated to the general population. However, refusal rates in these studies were < 1% and studies in other settings have highlighted that EAG studies, while biased, are able to provide a reasonable approximation of community-level malaria prevalence/burden and provide sufficient information to inform program activities [24]. Ideally, these results would be directly compared with community-level estimates to assess the degree of bias, but these data were not available at the time of publication. Similarly, the slightly different selection processes between venue types and study sites may have affected the results. However, the estimates of malaria burden are consistent with what is known for the area and expected based on the transmission biology. Surveys in EAGs can miss areas where there is no venue. This is a problem that concerns areas with low human density, since communities tend to have gathering venues such as schools and markets. Arguably, household surveys face the exact same issue—isolated areas with low human density are under-represented. Next, due to logistical constraints, only a subset of those testing negative by hsRDT were geolocated, only providing a fraction of the spatial coverage of the samples and expected population being represented. Random selection of negative individuals for household geolocation was performed to minimize the bias as much as possible. In addition, one of the inherent challenges with studying primary school-aged children is information bias. Children might not understand everything being asked or might not answer truthfully. We attempted to minimize this potential bias by inviting an older sibling (if available) to assist with responding to the questionnaire, and modified the EAG questionnaire for schools to ensure simple language and short length. Moreover, the information bias, if any, is unlikely to be different according to hsRDT status. Finally, this study was neither designed nor powered to assess risk factors for malaria in a low-transmission setting. However, associations with several factors were explored to better characterize the population at risk in different EAGs and to inform an upcoming case-control study to be completed in Grand’Anse. Despite these important limitations, we obtained good spatial representativeness of the venues and used the same questionnaire, instruments, protocols, and survey teams in both sites to ensure valid inferences could be made.

## Conclusions

We conducted surveys to estimate malaria prevalence in three different EAG populations in two regions of Haiti with different underlying malaria transmission intensity. Spatial and demographic variations in hsRDT prevalence were observed within and between each setting, offering increased spatial granularity of malaria transmission compared to routine confirmed malaria cases reported at the facility level and national surveys. In low-transmission settings like Haiti, EAG surveys provide a convenient alternative for targeted surveillance to identify non-care seeking and/or asymptomatic malaria infections below the health facility population catchment level. The increased granularity of information can then be used for data-driven decision-making and more tailored responses for reducing transmission and reaching the ultimate goal of malaria elimination. By identifying a malaria focus at an elevation of 650 m, this study notably found that malaria transmission was possible in areas usually not targeted by programmatic or modeling efforts.

## Supplementary information

**Additional file 1.** Summary Euclidean straight-line distance (meters) between household location and sampling venue for the subset of individuals with spatial coordinates available, according to hsRDT positivity by venue types (health facility, school) and study location (Artibonite, Grand’Anse).

**Additional file 2.** Mode of travel and self-reported time it took to arrive at the venue on the day of the study.

**Additional file 3.** Results comparison between cRDT and hsRDT, by region and type of venue.

## Data Availability

All anonymized data and R scripts used for this analysis can be made available by contacting the corresponding author.

## Abbreviations

AOR: Adjusted odds ratio
CI: Confidence interval
cRDT: Conventional rapid diagnostic test
DHIS: District health information system
EAG: Easy access group
GPS: Global position system
hsRDT: Highly sensitive rapid diagnostic test
IQR: Interquartile range
PCR: Polymerase chain reaction
WHO: World Health Organization
