# Supplementary material for: Programmatic options for monitoring malaria in elimination settings: easy access group surveys to investigate Plasmodium falciparum epidemiology in two regions with differing endemicity in Haiti
Source: BMC Med. 2020 Jun 23;18:141. doi: 10.1186/s12916-020-01611-z (PMC7310408; doi:10.1186/s12916-020-01611-z)
Supplement: Supplementary file 1 — Additional file 1. Summary Euclidean straight-line distance (meters) between household location and sampling venue for the subset of individuals with spatial coordinates available, according to hsRDT positivity by venue types (health facility, school) and study location (Artibonite, Grand’Anse). [file 12916_2020_1611_MOESM1_ESM.docx]

**Additional file 1** Summary Euclidian straight line distance (meters) between household location and sampling venue for the subset of individuals with spatial coordinates available, according to hsRDT positivity by venue type (health facility, school) and study location (Artibonite, Grand’Anse).

|  | **Artibonite** | | | | **Grand’Anse** | | | |
| --- | --- | --- | --- | --- | --- | --- | --- | --- |
|  | **Health Facility** | | **School** | | **Health Facility** | | **School** | |
| hsRDT Result | Pos | Neg | Pos | Neg | Pos | Neg | Pos | Neg |
| Min | 2510 | 12.7 | 325 | 5.76 | 6.0 | 9.17 | 20.5 | 8.99 |
| 25^th^ Quartile | 2854 | 658 | 1100 | 470 | 404 | 192 | 202 | 347 |
| Mean | 3588 | 3679 | 2824 | 1407 | 1691 | 1088 | 647 | 1169 |
| 75^th^ Quartile | 3225 | 4942 | 5320 | 1756 | 2183 | 1455 | 594 | 1158 |
| Max | 8907 | 31325 | 6086 | 14246 | 10370 | 10370 | 6731 | 11446 |
