# Supplementary material for: Programmatic options for monitoring malaria in elimination settings: easy access group surveys to investigate Plasmodium falciparum epidemiology in two regions with differing endemicity in Haiti
Source: BMC Med. 2020 Jun 23;18:141. doi: 10.1186/s12916-020-01611-z (PMC7310408; doi:10.1186/s12916-020-01611-z)
Supplement: Supplementary file 2 — Additional file 2. Mode of travel and self-reported time it took to arrive at the venue on the day of the study. [file 12916_2020_1611_MOESM2_ESM.docx]

**Additional file 2** Mode of travel and self-reported time it took to arrive at the venue on the day of the study.

|  | **Artibonite** | | | **Grand’Anse** | |
| --- | --- | --- | --- | --- | --- |
| N (%) | **Health Facility** | **School** | **Church** | **Health Facility** | **School** |
| Walk | 1054 (50.6) | 1911 (90.1) | 1519 (87.0) | 2091 (85.2) | 2480 (99.0) |
| Bicycle | 1 (0.05) | 4 (0.19) | 5 (0.28) | 9 (0.37) | 4 (0.16) |
| Local Transport -Boite/Hiace | 4 (0.19) | 47 (2.2) | 9 (0.51) | 2 (0.08) | 0 |
| Horse/Donkey | 8 (0.38) | 3 (0.14) | 0 | 9 (0.37) | 4 (0.16) |
| Public Transport – Bus/TapTap | 439 (21.1) | 62 (2.9) | 70 (4.0) | 7 (0.28) | 7 (0.28) |
| Motorcycle | 552 (26.5) | 82 (3.9) | 135 (7.7) | 331 (13.5) | 9 (0.36) |
| Car/Truck | 26 (1.2) | 12 (0.56) | 7 (0.40) | 4 (0.16) | 0 |
| Travel Time Summary (min) | | | | | |
| Min | 1 | 1 | 1 | 1 | 1 |
| Q1 | 10 | 7 | 5 | 5 | 8 |
| Median | 30 | 15 | 10 | 10 | 15 |
| Q3 | 60 | 30 | 15 | 30 | 30 |
| Max | 180 | 150 | 180 | 180 | 160 |
