# Supplementary material for: Programmatic options for monitoring malaria in elimination settings: easy access group surveys to investigate Plasmodium falciparum epidemiology in two regions with differing endemicity in Haiti
Source: BMC Med. 2020 Jun 23;18:141. doi: 10.1186/s12916-020-01611-z (PMC7310408; doi:10.1186/s12916-020-01611-z)
Supplement: Supplementary file 3 — Additional file 3. Results comparison between cRDT and hsRDT, by region and type of venue. [file 12916_2020_1611_MOESM3_ESM.docx]

**Additional file 3** Results comparison between cRDT and hsRDT, by region and type of venue

|  | **Artibonite** | | | | | | **Grand’Anse** | | | |
| --- | --- | --- | --- | --- | --- | --- | --- | --- | --- | --- |
|  | **Health Facility** | | **School** | | **Churches** | | **Health Facility** | | **School** | |
| **hsRDT**  **cRDT** | + | - | + | - | + | - | + | - | + | - |
| + | 25 | 0 | 15 | 0 | 5 | 0 | 266 | 0 | 75 | 0 |
| - | 1 | 2077 | 3 | 2101 | 1 | 1757 | 12 | 2232 | 2 | 2427 |
